# Epidémiology of malaria from 2019 to 2021 in the southeastern city of Franceville, Gabon

**DOI:** 10.1186/s12889-022-14765-7

**Published:** 2022-12-10

**Authors:** Judicael-Boris Lendongo-Wombo, Sandrine-Lydie Oyegue-Liabagui, Jean-Claude Biteghe-Bi-Essone, Edgard Brice Ngoungou, Jean-Bernard Lekana-Douki

**Affiliations:** 1 Unité Evolution, Epidémiologie et Résistances Parasitaires (UNEEREP), Centre Interdisciplinaire de Recherches Médicales de Franceville (CIRMF), P.O. Box 769, Franceville, Gabon; 2 Ecole Doctorale Régionale d’Afrique Centrale en Infectiologie Tropicale (ECODRAC), Franceville, Gabon; 3 Département d’Epidémiologie, Biostatistiques et d’Informatique Médicale (DEBIM)/Unité de Recherche en Epidémiologie des Maladies Chroniques et Santé Environnement (UREMCSE), Université des Sciences de la Santé (USS), Libreville, Gabon; 4 Département de Biologie, Faculté Des Sciences, Université Des Sciences Et Techniques de Masuku, Franceville, Gabon; 5 Département de Parasitologie-Mycologie, Université des Sciences de la Santé (USS), P.O. Box 769, Libreville, Gabon

**Keywords:** Malaria, Epidemiology, Seasonal variations, Franceville

## Abstract

**Background:**

In Gabon, a new national malaria control policy was implemented in 2003. It resulted in a decrease in the number of malaria cases in the country. In March 2020, the disruption of routine health services due to the COVID-19 pandemic has led to an increase in cases and deaths due to malaria. However, in Franceville, south-east Gabon, no data on malaria cases recorded before, during and after the COVID-19 epidemic has been published. Thus, the objective of this study was to determine the epidemiological characteristics of malaria in Franceville from 2019 to 2021.

**Methods:**

A retrospectively study of malaria cases was performed at the *Hôpital de l’Amitié Sino-Gabonaise* (HASG). Information regarding age, gender, malaria diagnosis by microscopy and hematology cell count were collected from laboratory registers from June 2019 to December 2021. Malaria data were analyzed and correlated with seasonal variations.

**Results:**

The data of 12,695 febrile patients were collected from the laboratory registers of the HASG, among which 4252 (33.5%) patients were found positive for malaria. The malaria prevalence was 37.5% in 2020 year. This prevalence was highest compared to the 2019 (29.6%) and 2021 (31.5%) year (*p* < 0.001). During the short rainy season (October to December), a large increase in malaria cases was observed all three year, from 2019 to 2021 (*p* > 0.05).

**Conclusion:**

The prevalence of malaria in Franceville was very high during COVID-19 pandemic. It is therefore necessary to strengthen existing interventions and implement more effective interventions.

## Background

Despite significant efforts to control and eradicate malaria, this deadly parasitic disease remains the most prevalent in the world. According to the World Health Organization (WHO) 2021 World Malaria Report, a total of 241 million malaria cases were recorded in 2020, representing a 12 million increase compared to 2019 (229 million cases) [1]. Approximately 627,000 malaria deaths were estimated to occur in 2020, 12% increase compared to 2019 (558,000 deaths) [1], due to the disruption of health services during the COVID-19 pandemic. Sub-Saharan Africa alone accounts for nearly 96% of all cases. Children under 5 years old, and pregnant women, remain the population groups with the highest risk of contracting malaria [2, 3].

In Gabon, a Central African country, the malaria transmission is stable throughout the year with mean prevalence of 22.3% (data of the National Malaria Control Programme). In the country, three plasmodial species: *P. falciparum, P. malariae* and *P. ovale* have been reported [4]. Among them, *P. falciparum* is the most widespread, (responsible for all cases of infection in some areas), followed by *P. malariae* (0.5-5%) and *P. ovale* (0.5-2%) [5–8]. In 2002, a new national malaria control policy was implemented to control malaria. It included artemisinin-based combination therapy (ACT), the use of long-lasting insecticidal nets (LLINs),the use of intermittent preventive treatment with sulfadoxine-pyrimethamine (IPTp-SP), which has a protective effect in the mother and fetus, and finally, early diagnosis of all suspect cases in order to initiate care [1]. These measures led to a decrease in the burden of malaria infection in the urban and rural areas.In Libreville and Franceville (urban areas), prevalence of malaria infection decreased from 31.2 to 18.3% [9] and from 69 to 19.5% [10], respectively. The decreased in malaria prevalence has led to the age of the populations at risk of malaria shifted in Libreville and Franceville [9, 10]. The intensification of malaria control measures in children under 5 years of age has resulted in low exposure to *Anoheles* bites. As a result, a delay in the development of immunity to malaria has been observed, putting children over 5 years of age at risk of malaria [9, 10]. In Makokou and Dienga (rural areas of Gabon), the malaria prevalence were 54.4% and 43.5% respectively [11, 12]. In general, the prevalence of malaria in rural Gabon is poorly documented. In Gabon, the COVID-19 pandemic was declared in March 2020. This led to the implementation of restrictive measures from 6pm to 5am and a disruption of health services. This dysfunction of the health services has led to an increase in cases and deaths due to malaria. However, in Franceville, south-east Gabon, no data on malaria cases recorded before, during and after the COVID-19 epidemic has been published. Thus, the objective of this study was to determine the epidemiological characteristics of malaria in Franceville from 2019 to 2021.

## Methods

### Study site and patients

This study was conducted at the HASG in Franceville (in Haut-Ogooué Province, Gabon), an urban area in southeastern of Gabon, with a population of 129,694 people. This retrospective study involved all patients who came at the hospital for fever or a history of fever in the last 48 h (base axillary temperature or rectal temperature > 37.5 °C).

### Data collection

Socio-demographic information such as age and gender, biomedical information such as blood cell count and malaria diagnostic result of all patients were collected from laboratory registries from 1 to 2019 to 31 December 2021.

The consultation of the laboratory registers was carried out in accordance with the Declaration of Helsinki 2000. This study was approved by the National Research Ethics Committee of Gabon (N°0021/2022/CNER/P/SG) which exempts the patient’s informed consent in the context of a retrospective study and by the hospital management, which is the guarantor of hospital data (1845/MS/SG/DRSSE/HASG). The data were collected anonymously as part of the routine analyses performed at the laboratory of the HASG, this health structure is supervised by a Director.

At the HASG, light microscopy (LM) is used in clinical practice to detect malaria infection in all patients presenting to health facilities with symptoms. Thick blood smears were defined as positive if any asexual forms of *Plasmodium sp* were observed by at least two microscopists and parasitaemia are calculated based on WHO guidelines [12]: low (+) 1–10/100 fields, mild (++) 11–100/100 fields, moderate (+++) 1–10/one field, and high parasitaemia (++++) > 10/one field. Unfortunately, information on the *Plasmodium* species diagnosed is not available in the registers. Nevertheless, *P. falciparum* accounts for more than 94% of the malaria infection [5, 10]. Severe anemia was defined as a hemoglobin value < 5 g/dL in children and < 7 g/dL in adults [13].

### Data analysis

Data of all patients were recorded in Excel 2013 spreadsheets. Statistical analyses were carried out with Epi-info version 3.3.2 (2005, CDC, Atlanta, USA) and the R software version 4.0.5 (2021-03-31). Patients with missing data were excluded for the analysis. Qualitative variables have been described by proportion and quantitative variables by mean, standard deviation (SD), median with inter-quartile range (IQR). Proportions were compared using the Chi2 test. For all analyses, the significance level was set at α = 5%.

## Results

### Sampling description

**Fig. 1:**
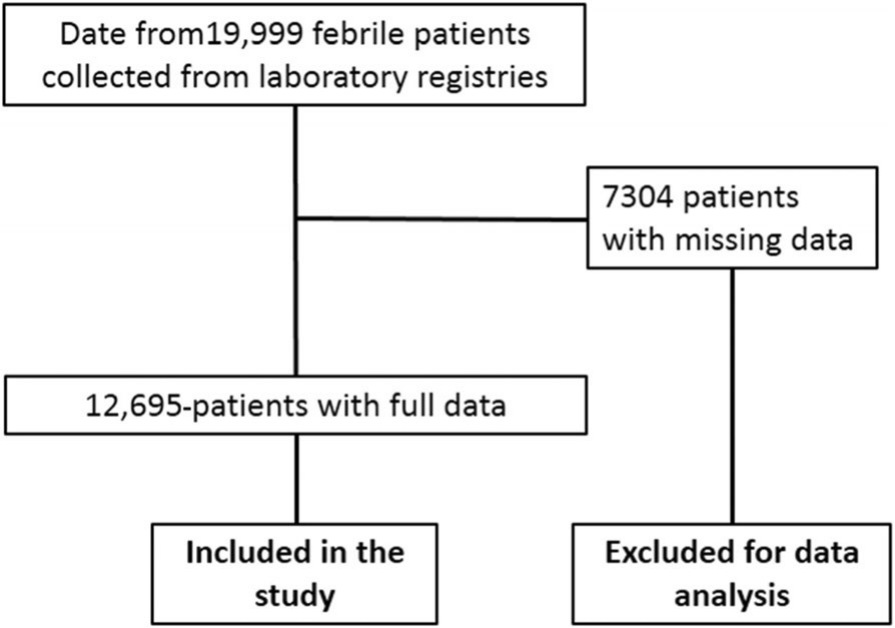
Flowchart of enrolment for patient

The data of 19,999 febrile patients were collected from laboratory registries and 7304 of them had missing data. Febrile patients with full data included in the analysis are shown in Fig. 1.

In total, data of 12,695 febrile patients were analysed, 4881 males and 7814 females with a gender ratio (M/F) of 0.64. Two thousand four hundred and nine (2419), 4977 and 5299 patients were included respectively for 2019, 2020 and 2021. The average age of the patients was 19.8 ± 19.4 years, 23.3 ± 20.3 years and 23.6 ± 21.6 years for 2019, 2010 and 2021 respectively (*p* < 0.001). Children aged between 0 and 5 year old represented 29.41% (3734/12,695) of the patients, and patients over 5 year old 70.59% (8961/12,695). Table 1 shows the general demographic and clinical characteristics of all patients. Forty-nine (0.39%, 49/12,695; 95% CI [0.29–0.51]) patients had severe anemia. Means Hematological values for White blood cells, red blood cells and platelets were normal.

**Table 1:** Demographic and clinical characteristics of patients

General characteristics	
***N*** (number)	12,695
**Age** (year)	
- Mean age (SD)	22.7 (20.7)
- Median [IQR]	18 [4—37]
**Age groups stratified**	
- [0–5], n (%)	3734 (29.41)
- [6–10], n (%)	1325 (10.43)
- [11–15], n (%)	955 (7.52)
- [16–49], n (%)	4979 (39.22)
- > 49, n (%)	1702 (13.41)
**Hemoglobin (g/dL)**	
- Mean age (SD)	11.1 (2.02)
- Median [IQR]	11.1 [9.80—12.3]
**White blood cells (× 10**^**3**^**/μl)**	
- Mean age (SD)	6.86 (3.90)
- Median [IQR]	6 [4.80—8]
**Red blood cells (× 10**^**6**^**/μl)**	
- Mean age (SD)	4.96 (13.4)
- Median [IQR]	4.37 [3.94—4.80]
**Platelets (× 10**^**3**^**/μl)**	
- Mean age (SD)	356 (172)
- Median [IQR]	336 [238—446]

### Characteristics of malaria infection

In total, 4252 patients were positive for malaria infection, representing a prevalence of 33.49% (4252/12,695, 95% CI [32.68–34.32]) with the mean age of 18.98 ± 20.72 years old. The malaria prevalence was higher in 2020 (37.5%, 1866/4977; 95% CI [36.16–38.85]) than 2019 (29.6%, 717/2419; 95% CI [27.85–31.49]) and 2021 (31.5%, 1669/5299; 95% CI [30.26–32.76]), *p* < 0.001. Among the 4252 positive patients, 15 (0.35%, 95% CI [0.21–0.58]) had severe anemia. Patients over 12 years old account for 73.33% (11/15) of them. Hemoglobin Characteristics of infected malaria patients by year are shown in Table 2. The mean hemoglobin level of malaria patients were was higher in 2020 (10.1 ± 2.1) compared to other years (*p* = 0.01). In general, patients with *Plasmodium* infection had moderate anemia.

**Table 2:** Hematological parameters of malaria patients

	Mean ± standard deviation			*p*
	2019	2020	2021	
Number	717	1866	1669	
White blood cells (x 1000/mL)	8.2 ± 3.6	7.3 ± 3.2	7.5 ± 4.0	0.3
Red blood cells (x 1000 m/mL)	3.4 ± 1.0	4.1 ± 1.0	4.9 ± 17.4	0.07
Platelets (x 1000/mL)	280.7 ± 129.3	302.3 ± 171.7	404.2 ± 213.9	**< 0.001**
Hemoglobin (g/dL)	8.4 ± 1.6	10.1 ± 2.1	9.4 ± 2.2	**0.01**
Hematocrit (g/dL)	26.1 ± 5.2	32.1 ± 6.5	29.9 ± 6.9	< **0.001**
Average blood volume (fL)	102.5 ± 127.4	79.2 ± 11.4	77.5 ± 10.6	**< 0.01**
Average corpuscular hemoglobin tenor (pg)	24.6 ± 2.7	25.5 ± 5.7	24.9 ± 11.7	**< 0.001**
Mean corpuscular hemoglobin concentration (g/dL)	32.3 ± 1.7	31.5 ± 2.3	31.3 ± 3.5	**< 0.001**

### Seasonal distribution of malaria by year

**Fig. 2:**
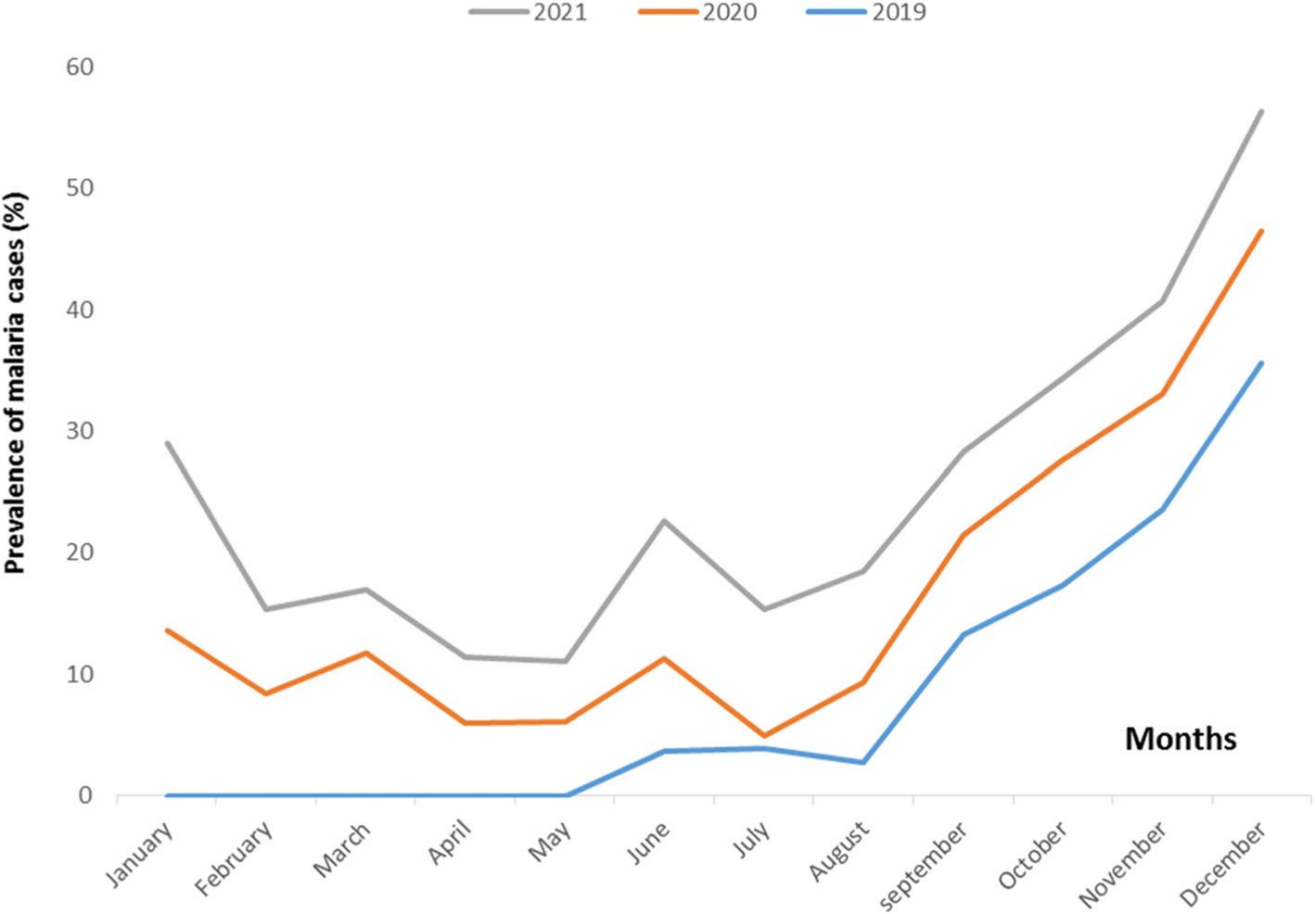
Temporal distribution of malaria infection

Figure 2 shows the distribution of malaria cases in for years 2019, 2020, and 2021. This distribution of malaria cases varies from year to year, and according to season in a given year. During the short rainy season (October - December), there was an increase in malaria cases during the three years of the study. However, no significant difference was observed between the years2019, 2020 and 2021 during the short rainy season (*p* = 0.148). During the long rainy season (April - June), a lower malaria prevalence was observed in 2019 (13.2%) and 2021 (17.4%), compared to the short rainy season. The variation in the number of malaria cases during the long rainy season showed significant differences between the different years (*p* < 0.01). Morever, during the long dry season and the short dry season, the variation of malaria prevalence was significantly different between the years 2019, 2020 and 2021 (*p* < 0.01). Data for the first four months (January, February, March, and April) of 2019 were missing from laboratory records.

### Distribution of confirmed malaria cases according to age group in 2019, 2020 and 2021

**Fig. 3:**
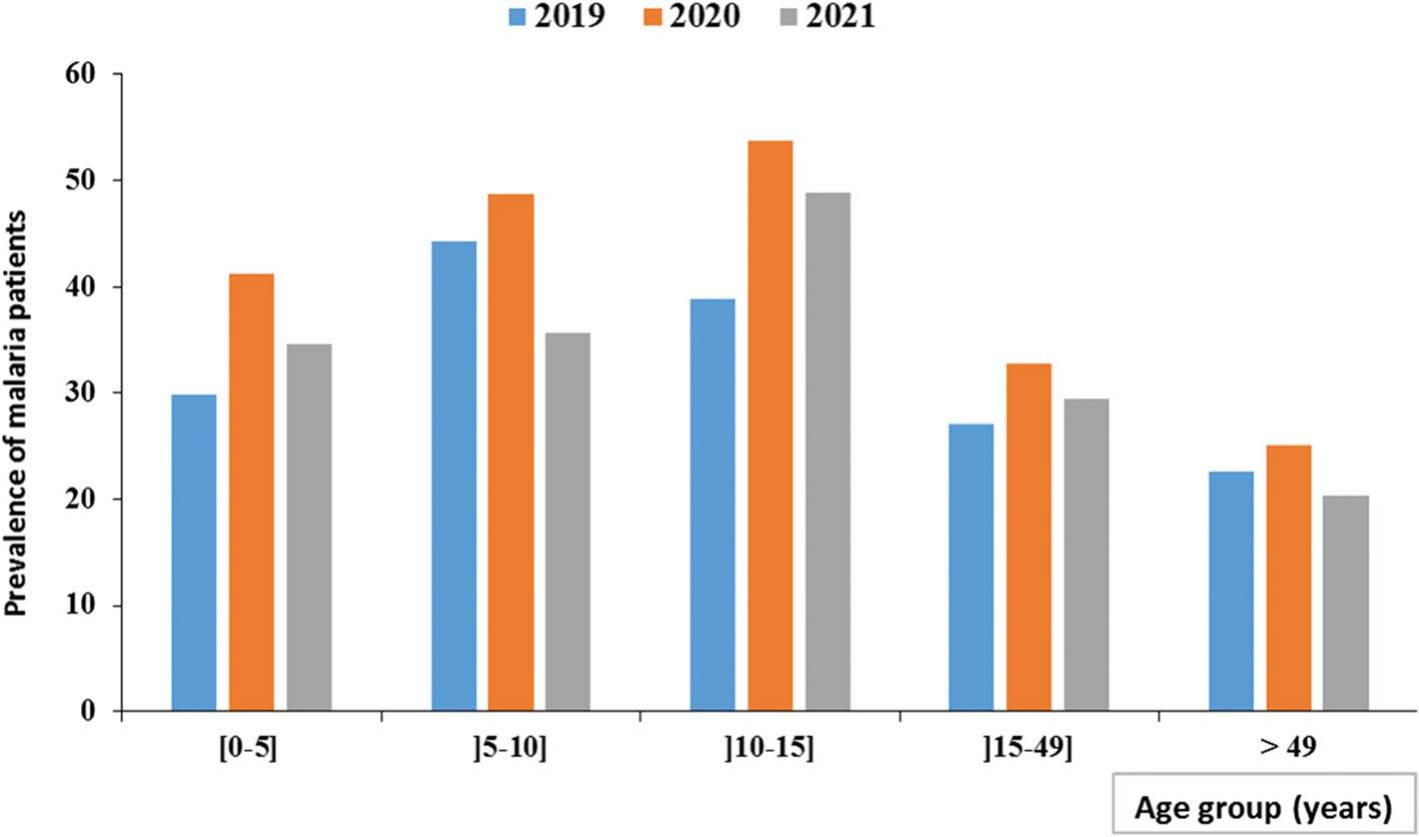
Distribution of confirmed malaria cases by age group and age in 2019, 2020 and 2022

Pairwise comparisons showed that this change in malaria cases was significantly different between 2019 and 2020 (*p* < 0.001), 2020 and 2021 (*p* < 0.0001), 2019 and 2021 (*p* < 0.001). Figure 3 shows that the year 2020 has the highest prevalence of malaria infection in all age groups. In 2019, malaria infection was most prevalent (44.3%) in the [6–10 years] age group. In 2020 and 2021, malaria was most prevalent in the [11–15 year] age group, with prevalence of 53.8% and 48.9%, respectively. In the age groups [0–5 years] (*p* < 0.0001); [6–10 years] (*p* < 0.001); [11-15years] (*p* < 0.01) and [16-49years] (*p* < 0.01), the prevalence of malaria infection differed significantly from one year to the next.

## Discussion

During the 2000s, published data on malaria epidemiology across sub-Saharan Africa showed a decline in malaria prevalence [14–16]. Several studies conducted in Gabon also describe a decline in malaria cases between 2005 and 2010 in urban and semi-urban areas [9, 17]. This decrease in malaria prevalence is the result of the implementation of a malaria control policy in Gabon starting in 2002, including intermittent preventive treatment of pregnant women, distribution of insecticide-treated nets (ITNs), and increased availability of effective antimalarial drugs [9, 18]. After 2010, studies describe a resurgence in the number of malaria cases in both rural and urban areas of Gabon [18–20]. In this study, the malaria burden in Franceville was 33.5% for the 2019–2021 period. This high prevalence of malaria could be explained by the lack of awareness campaigns and the absence of vector control methods during that time [11]. This prevalence of malaria infection is higher than those described in 2010 (17.9%) by Lekana-Douki et al., and in 2013 (21.2%) by Maghendji-Nzondo et al. [5, 10]. This study showed that the malaria prevalence is higher in 2020 during the COVID-19 pandemic compared to 2019 and 2021, before and after COVID-19 pandemic respectively. These results are in accordance with global data that showed an increase in malaria cases during the COVID-19 pandemic [1]. The implementation of pandemic’s restrictive measures has led to the disruption of healthcare infrastructure, to a decrease in the finance of households (lack of financial resources to buy antimalaria in drugstores) and consequently to a decrease in the malaria control.

In sub-Saharan Africa, children under 5 years of age have often been the age group who is most at risk of developing malaria [21]. In this study, the age group [6–9] years had the highest malaria prevalence (44.3%) in 2019 while the age group [10–14] years had the highest malaria prevalence in 2020 and 2021 (53.8% and 48.9% respectively). These results may indicate a shift in the age group of patients at risk for malaria infection in accordance with study carried-out in Cameroon [22] or may reflect broader trends in higher prevalence among older children [23, 24]. Also, this result could be explained by the fact that during the COVID-19 pandemic, adolescents in this age group spent more time outside the home due to the idleness created by the COVID-19 restrictive and were therefore more exposed to mosquito bites. It has been recognized for more than a decade that measures which reduce the level of exposure may interfere with the natural acquisition of immunity to malaria[25]. This shift could show a delay in the acquisition of malaria immunity among adolescents and the factors associated with this delayed immunity acquisition remain to be elucidated [26, 27]. For each age group, the prevalence of malaria was higher in 2020. This result could be explained by the negative impact of COVID-19 on the epidemiology of malaria causing disruptions in health services, affecting the implementation of prevention and treatment policies, which are reflected in an increase in the number of malaria cases during the 2020 year [28]. This study shown low prevalence (0.39%) for severe anemia. This prevalence seems to be lower than that found by Maghendji-Nzondo in Franceville, at the Centre Hospitalier Régional Amissa Bongo (CHRAB) and Dzeing-Ella in Libreville [5, 29]. This result could be explained by the fact that in the HASG of Franceville, all critical clinical cases are systematically transferred to the CHRAB which is the reference health structure in the Haut-Ogooué province, for better management.

The annual prevalence of malaria for the years 2019, 2020 and 2021 were 30.2%, 37.4% and 31.5%, respectively. The variation in malaria infection during the long rainy season, long dry season, and short dry season was significantly different between 2019, 2020 and2021 (*p* < 0.01). For all three years of the study, malaria was most prevalent during the short dry season. This result could be explained by the abundance of Anopheles mosquito breeding sites during this period of the year, since abundant rainfall makes it the wettest season. This result is also consistent with those of Maghendji-Nzondo which showed a higher prevalence of malaria cases during the short rainy season, with prevalence of 26.4% in 2011 and 29.8% in 2012 [5, 30]. Similarly, other studies conducted in Gabon and other areas have shown that malaria infection prevalence are higher during the short rainy season [31, 32]. This study provides necessary data for the epidemiological surveillance of malaria and its use by policy-makers.

This study has a few limitations. Data gathered on malaria infection were based on hospital laboratory registries and there were missing data. There could be selection bias (hospital-based), no species diagnosis has been performed for *Plasmodium* infection and the absence of data on severe malaria. Also, data of several patients were missing and could not be included in analyses. In addition, this study did not include data from rural areas due to difficulties in accessing these data (remoteness, absence or low conservation of hospital registers, lack of malaria diagnostic equipment) and limited funding.

## Conclusion

In this study, we observed a high prevalence of malaria in hospital in Franceville from 2019 to 2021. We observed a shift in the age group who is most at risk of malaria older children. It is therefore necessary to maintain and extend malaria control measures to all age groups and in all regions of the country to provide a better response to malaria infection.

## Data Availability

The datasets used and/or analyzed during the current study available from the corresponding author on reasonable request.
